# Morphological characterization and effect of diatomaceous earth on the cure characteristics and tensile properties of natural rubber vulcanizates

**DOI:** 10.1039/d5ra05031f

**Published:** 2025-09-30

**Authors:** N. B. J. De Costa, S. M. Egodage, M. Maddumaarachchi

**Affiliations:** a Department of Polymer Science, Faculty of Applied Sciences, University of Sri Jayewardenepura Sri Lanka madhubh@sjp.ac.lk; b Department of Chemical and Process Engineering, University of Moratuwa Sri Lanka

## Abstract

This study aims to discover the fundamental morphological properties of Diatomaceous Earth (DE) and its impact on the curing characteristics and primary tensile properties of natural rubber vulcanizates to substitute commercial silica. The methodology involved detailed characterization of DE and commercial silica using Scanning Electron Microscopy (SEM), Dynamic Light Scattering (DLS), X-ray Diffraction (XRD), X-ray Fluorescence Spectroscopy (XRF), and Fourier-Transform Infrared Spectroscopy (FTIR). Rubber composites were formulated using 55 parts per hundred parts of rubber (phr) of DE and silica, incorporating DE in proportions of 25%, 50%, and 75% of filler by weight. Cure properties were determined using a Moving Die Rheometer (MDR) at 150 °C, and ultimate tensile strength was assessed with a Universal Testing Machine. The findings revealed that the 100% DE-filled composite demonstrated poor curing characteristics, evident from its low cure rate index and reduced cross-link density. Composites containing 25% DE blended with silica exhibited superior ultimate tensile strength compared with those containing only silica as a filler, attributed to the advantageous synergistic effect arising from particle size and the enhanced surface area of DE. Nonetheless, loadings exceeding 25% of DE resulted in diminished tensile properties due to low density of the DE filler. These discoveries may facilitate the implementation of DE as a bio-based material in polymer composites.

## Abstract 2

Partial or complete replacement of silica with bio-based diatomaceous earth (DE) in natural rubber composites maintains mechanical properties, offering a pathway for eco-friendly rubber materials.

## Graphical abstract

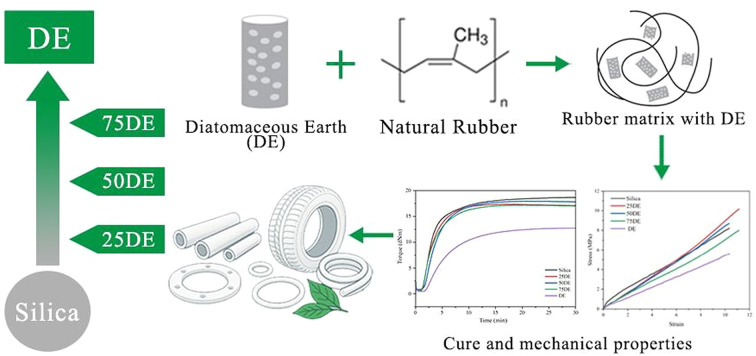

## 1 Introduction

Commercial silica is one of the most widely used reinforcing fillers in the rubber industry. Silica mainly comprises SiO_2_ and it is amorphous. However, silica, being an inorganic material, shows low compatibility with the organic rubber matrix. Hence, coupling agents such as bis(triethoxysilylpropyl)-tetrasulphide (TESPT) are added to silica-filled rubber composites in order to get optimum properties.^1,2^ Commercial silica is commonly employed for a wide range of specialty products such as solid and pneumatic tyres, gaskets, and non-marking tyres where the rubber product should be white in color. Though commercial silica is very versatile, the employment of bio-based silica fillers to replace synthetic silica is currently being studied to some extent. Bio-based silica fillers have received greater interest as the world slowly moves towards the green concept. Rice husk powder, silica extracted from rice husk, illite clay, and sepiolite can be identified as some such bio-based silica fillers.^3–7^

The effects of silica-based fillers on the cure characteristics, mechanical properties, and morphology of rubber composites have been investigated in some depth.^3–7^ These studies reveal that the processing method and interfacial interaction forces between the filler and the rubber matrix are the key to the mechanical properties of these composites. Silica extracted from rice husk has shown acceptable reinforcing capabilities, which has offered enhanced mechanical strength and sustainability as a bio-based filler;^4^ however, the extraction process is complex and energy-intensive, limiting its large-scale applicability and sustainability. Wang *et al.* found out that the surface modification of mineral-based fillers including illite clay using cationic surfactants could significantly improve filler dispersion and further enhances filler-rubber matrix interactions, which results in improved curing kinetics, crosslink density, and mechanical properties, with a 71.88% increase in MOD300 and reduced abrasion volume compared to unmodified fillers.^6^ Likewise, Tagliaro *et al.* discovered that incorporating silica with a combination of anisotropic fillers such as sepiolite forms a filler network inside the rubber matrix, supporting self-assembly and improving dynamic-mechanical properties such as reduced hysteresis and rolling resistance, which are vital for tyre applications.^7^

Diatomaceous earth (DE) is the fossilized exoskeleton of unicellular aquatic organisms called diatoms. DE contains a notable quantity of SiO_2_ with relatively small percentages of other oxides such as Al_2_O_3_, Fe_2_O_3_, and CaO in their composition alongside some minor elements such as Na, Mg, Mn and Ti.^8^ Since it is a naturally occurring material, its composition can vary depending on the geographical source and formation conditions. DE has a high fusion point and low heat conductivity.^9^ Due to the nanoporosity and low level of toxicity, DE is often used as a purifying agent for water and juices. DE is also utilized in detergents, deodorizers, and filter systems for swimming pools, and is employed as an anti-caking agent and animal feed additive.^10^ Though DE contains a significant amount of silica, to the best of our knowledge, its potential as a silica source in composites has only led to a few investigations.^11–15^

Factors such as the presence of impurities, particle size, and shape of filler materials can affect the properties of filled rubber composites.^16–19^ DE is a highly porous and low-density material compared to silica; therefore, the rheological behavior and primary mechanical properties of DE-filled natural rubber vulcanizates may differ from those of vulcanizates filled with silica. Nonetheless, if the synthetic silica could be replaced using DE, it would be highly beneficial in terms of sustainability. From an economic and environmental perspective, substituting synthetic silica with DE offers additional advantages. Economically, DE requires less intensive processing than commercial precipitated silica, reducing material costs and making DE-containing rubber compounds more attractive for industrial applications. Environmentally, DE production consumes less energy, generates lower CO_2_ emissions, and relies on a naturally occurring material, potentially decreasing the ecological footprint of rubber products. These considerations further justify the use of DE as a sustainable filler in rubber composites.

This research presents a comprehensive investigation into the influence of DE as a filler on the rheological behavior and key mechanical properties of natural rubber-based vulcanizates, a topic that has not been thoroughly explored in previous studies.

## 2 Experimental

### 2.1 Materials

DE (ground, 100% natural, food grade, freshwater type, BET specific surface areas of 34.500 m^2^ g^−1^)^20^ was purchased from Southern Homewares, South Carolina, United States, and the commercial silica and the coupling agent bis(triethoxysilylpropyl)tetrasulfide (TESPT) were obtained from a local supplier. The crepe rubber was obtained from the Industrial Development Board, Sri Lanka. Zinc oxide (ZnO), polyethylene glycol (PEG 400), polyethylene (PE) wax, stearic acid, 2,2,4-trimethyl-1,2-dihydroquinoline (TMQ), sulfur, dibenzothiazyl disulfide (MBTS), tetramethylthiuram monosulfide (TMTM), and *N*-(cyclohexylthio)phthalimide (CTP) as a pre-vulcanizing inhibitor, were purchased from Glorchem Lanka PLC. All chemicals and materials except DE and silica were used as received.

### 2.2 Methodology

#### 2.2.1 Characterization of fillers

Silica and DE were heated at 150 °C to eliminate moisture. Scanning electron microscopy (SEM) was performed to investigate the topography and morphology of silica and DE using a CARL ZEISS EVO 18 instrument operated at an acceleration voltage of 10 kV. Prior to imaging, the samples were sputter-coated with a thin layer of gold to enhance conductivity and image quality. Nicolet iS10 Fourier-transformed infrared spectrometer (FTIR) with the attenuated total reflectance (ATR) method was used to examine the functional groups. Spectra were recorded in the range of 4000–400 cm^−1^ with a resolution of 4 cm^−1^. The Fritsch-Analysette 22 NanoTec particle size analyzer, which uses dynamic light scattering (DLS) to measure particle size, was utilized to measure particle size in the range of 0.08–2100 μm. The crystallinity of the particles was assessed using a CuKα radiation source with a wavelength of 1.54178 nm and a 40 mA current power set on a Rigaku Ultima IV X-ray diffraction (XRD) machine operating in the 5° to 80° range. The elemental composition of DE was determined using a HORIBA Scientific XGT-5200 X-ray Fluorescence (XRF) microscope. Approximately 50 mg of sample in powder form was mounted on a clean sample holder. For each sample, six different areas (100 μm × 100 μm each) were selected and analyzed to account for potential sample heterogeneity.

#### 2.2.2 Preparation of rubber composites

##### 2.2.2.1 Compound formulation

The basic compound formulation used in this study is presented in Table 1. To explore the potential of replacing commercial silica with DE, several compound formulations were prepared, as shown in Table 2. The selected formulation represents a typical silica-filled rubber compound, incorporating TESPT as the coupling agent, PEG 400 and PE wax as processing aids, ZnO and stearic acid as vulcanization activators, TMQ as the antioxidant, sulfur as the vulcanizing agent, MBTS and TMTM as accelerators, and CTP as the pre-vulcanization inhibitor.

**Table 1:** Compound formulation

Ingredient	Amount (phr)
Crepe rubber	100.0
Filler	55.0
TESPT	6.9
PEG 400	2.7
PE wax	8.0
ZnO	5.0
Stearic acid	2.0
TMQ	2.0
Sulphur	2.5
MBTS	1.0
TMTM	0.1
CTP	0.1

**Table 2:** Compound formulation with varying silica and DE amounts

Compound	Silica amount (phr)	DE amount (phr)
Silica	55.00	0
25DE	41.25	13.75
50DE	27.50	27.50
75DE	13.75	41.25
DE	0	55.00

##### 2.2.2.2 Mixing process

The mixing was done in two stages. A laboratory-scale plastomill was used for the mixing process. The rotor speed was set at 60 rpm. Initially, the crepe rubber was added, and the rubber was masticated for 3 minutes under 50 °C. Then half of the filler was added along with the half of the TESPT coupling agent and was mixed for 5 minutes followed by the addition of the remaining half of the filler and the coupling agent. Then the temperature cranked up to 150 °C for 20 seconds and immediately, the temperature was reduced back to 50 °C while mixing. ZnO, Stearic acid, TMQ, PEG 400 and PE wax were added, and the mixing was done for 3 minutes. The sample was then removed and allowed to cool for 2 hours. The 2nd stage mixing was done for another 5 minutes along with the addition of sulfur, MBTS, TMTM, and CTP. The rubber lumps were processed into a sheet using a laboratory two-roll mill.

##### 2.2.2.3 Testing rheological behavior and tensile properties

The rheographs were obtained using the EKTRON EKT-2001S moving die rheometer at 150 °C. ASTM D412 standard was used (Die-E) to prepare the dumbbell shaped test specimens with a thickness of 2 mm, gauge length of 33 mm, gauge width of 6 mm, and overall length of 115 mm.^21^ The tensile properties were measured using the MARX Test universal tensile testing machine at a strain rate of 500 mm min^−1^.

## 3 Results and discussion

### 3.1 Characterization of fillers

The characterization of silica and DE disclosed significant insights regarding the morphological and topological attributes of these fillers. DE typically exists in two forms: disk and rod. DE in rod form is commonly used as an anti-caking agent, insecticide, and for various household applications. The disk-shaped DE is primarily utilized in the filtration process because of its distinctive sieve-like structure and nano-porosity.^8^

**Fig. 1:**
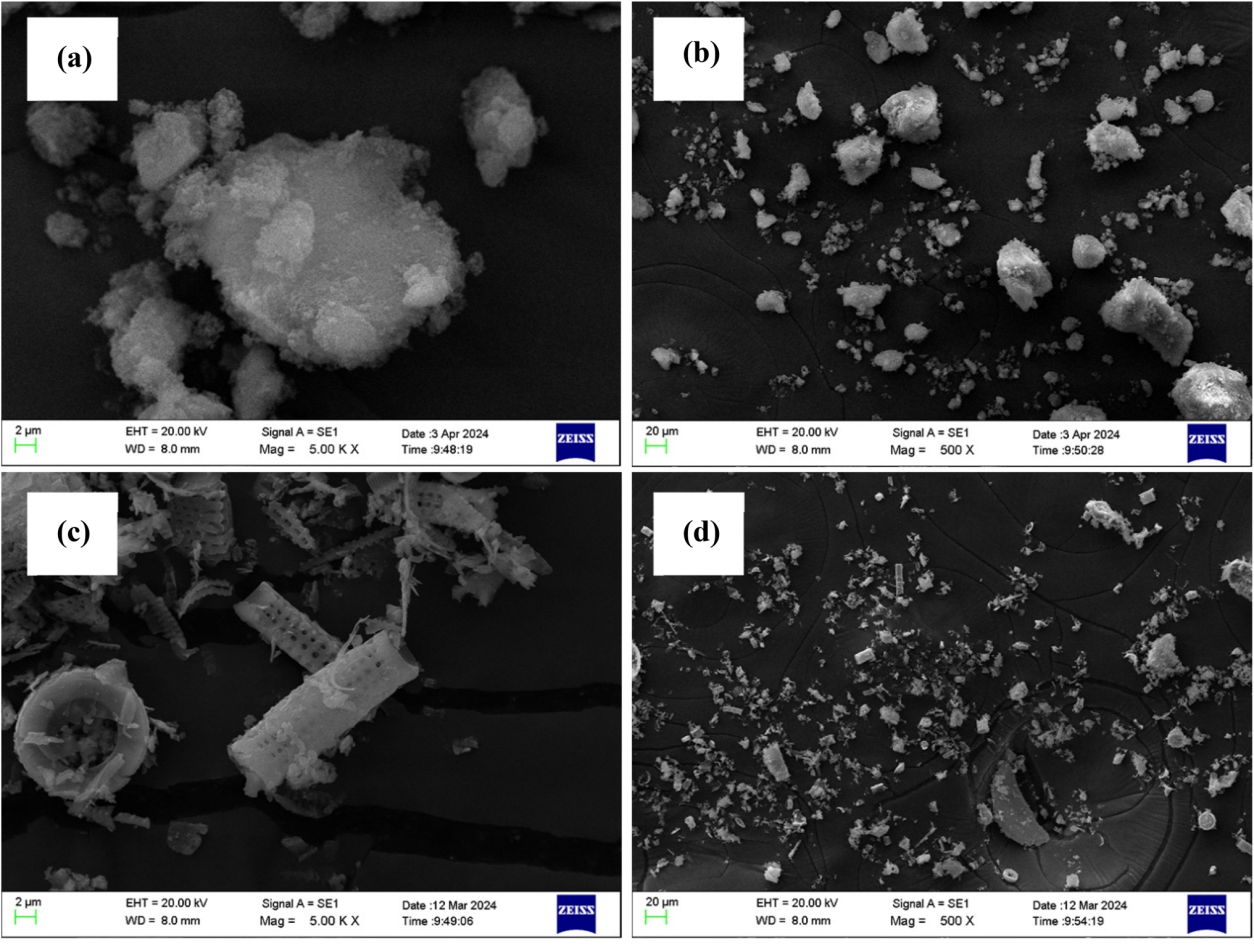
(a) SEM image of silica 5k× (b) SEM image of silica 500× (c) SEM image of DE 5k× (d) SEM image of DE 500×.

Fig. 1(a)–(d) illustrate the SEM images of silica and DE at 500× and 5k× magnifications. As shown in Fig. 1(b) and (d), which illustrate the particle sizes at 500× magnification, there is a clear difference between the particle sizes of silica and DE. Silica exhibits a considerably larger average particle size, and a broader particle size distribution compared to DE. It is further evident that DE is in rod shape and silica particles don't have a uniform shape.

**Fig. 2:**
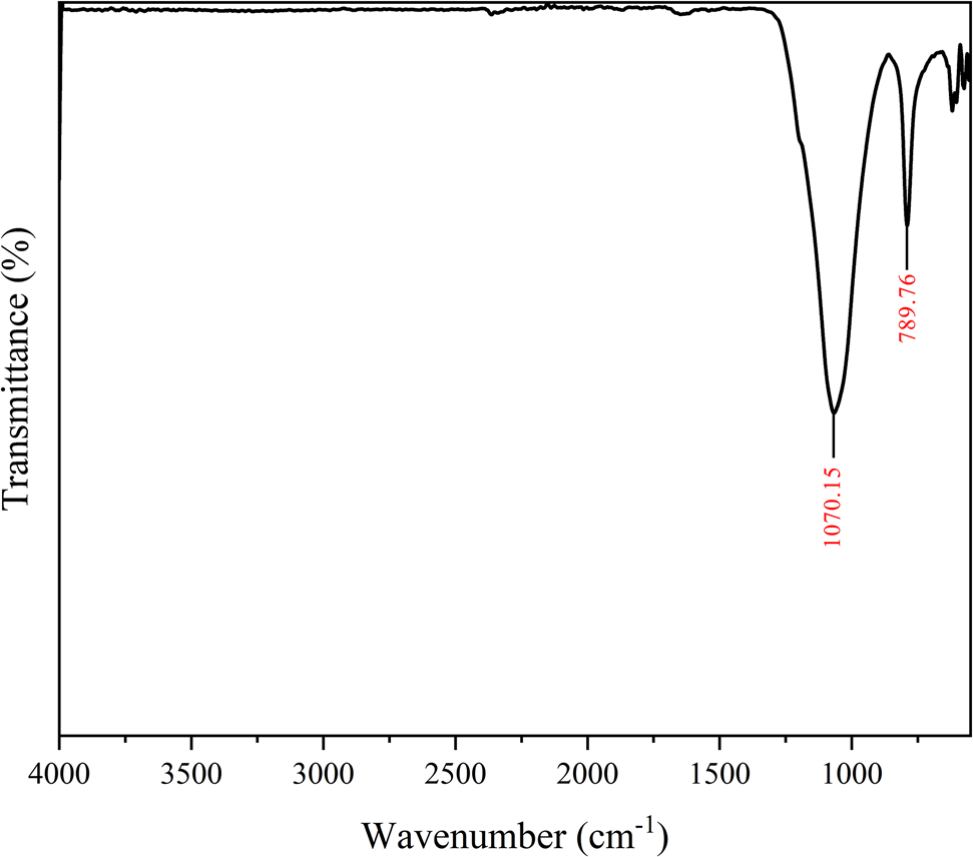
FTIR spectrum of DE.

Fig. 2 shows the FTIR spectrum of DE. The spectrum confirms the presence of the Si–O–Si stretching in the 1060–1080 cm^−1^ range and the Si–OH stretching in the 780–795 cm^−1^ region.^22^ The absence of additional functional groups highlights the purity of the DE particles.

**Fig. 3:**
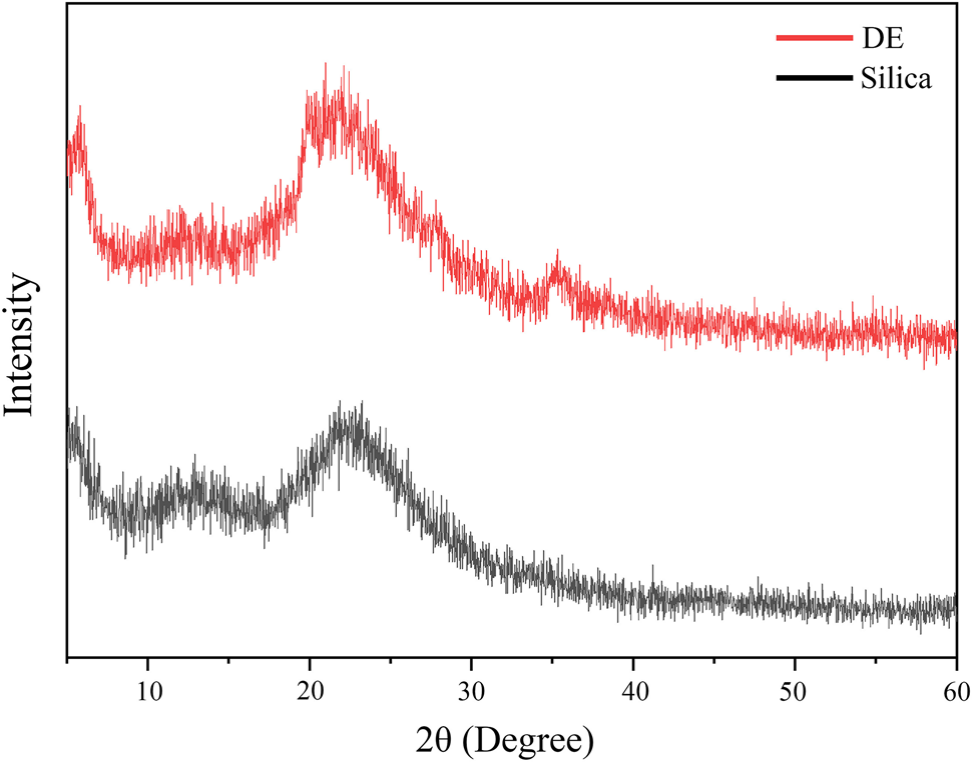
XRD of DE and silica.

XRD results shown in Fig. 3 revealed that the most prevalent morphology of both silica and DE is amorphous. The crystalline structure of the cristobalite-alpha primary structure of silica cannot be observed in these diagrams because of the amorphous nature.^8^

Table 3 presents the elemental composition of DE as determined by XRF analysis. The results show that the primary component among the detectable elements is silicon (Si), constituting 92.51% by weight, confirming that DE is highly silica-rich. Iron (Fe) is present at 3.36%, followed by calcium (Ca) at 1.82%. Minor components include titanium (Ti), potassium (K), sulfur (S), and aluminum (Al). This high silicon content highlights the suitability of DE as a potential replacement for commercial silica in various applications, offering a naturally occurring and cost-effective alternative. Most of these elements are naturally present in DE in the form of their respective oxides.^23^ Even at low concentrations, these oxides can influence the behavior of composites during processing and curing, although their effects are expected to be minimal given the small amounts detected in this sample.^24,25^

**Table 3:** Elemental compositions of DE obtained from XRF analysis

Element	Percentage^a^ (wt%)
Si	92.51 ± 0.31
Fe	3.36 ± 0.13
Ca	1.82 ± 0.06
Ti	0.82 ± 0.06
K	0.55 ± 0.05
S	0.53 ± 0.29
Al	0.43 ± 0.01

a The elemental percentages are given as a percentage of the total detected elements and not the actual elemental composition.

Table 4 provides the average particle size data for silica and DE, measured using DLS. The average size of silica is significantly larger compared to DE confirming the observations made with SEM. Furthermore, silica consists of a larger particle size distribution as evident in the SEM observations.

**Table 4:** Average particle size data from DLS

Sample name	Average particle size (μm)
Silica	26.39 ± 1.57
DE	10.07 ± 0.45

In summary, it is evident that silica and DE both are amorphous, and the particle size of the silica is higher than that of DE.

### 3.2 Rheological behavior and tensile properties

The rubber compounds were subsequently evaluated for their rheological properties and tensile properties. The rheological properties refer to the flow behavior of the material, which influences the morphology of the compound. These properties are critical, as they determine the vulcanization time and influence the final mechanical strength of the rubber. Tensile properties, on the other hand, provide a direct measure of its strength.

**Fig. 4:**
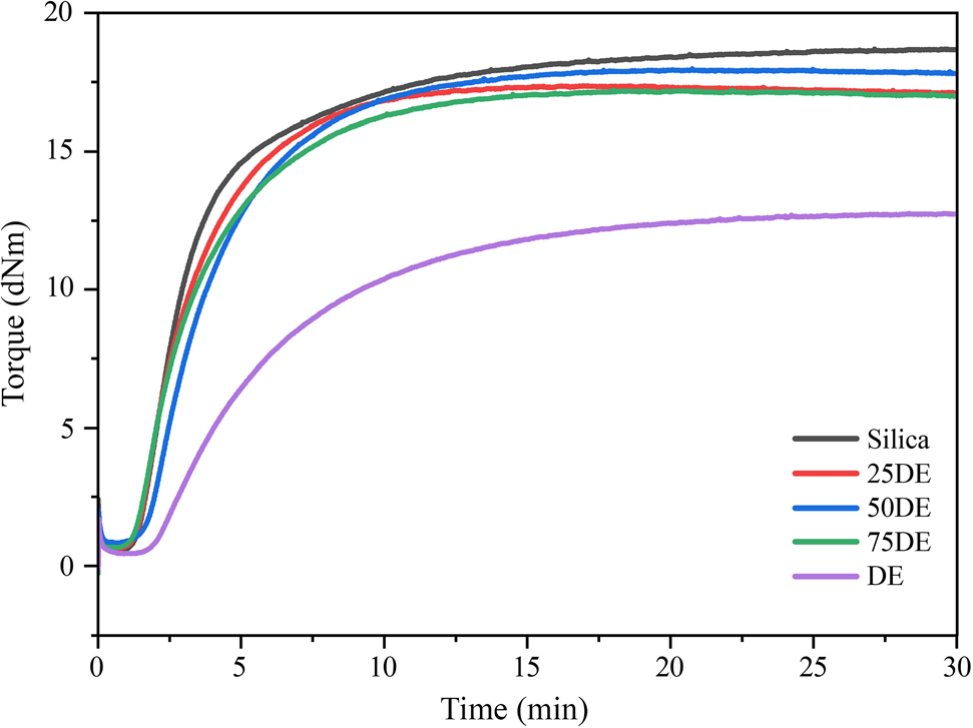
Cure characteristic curves of prepared rubber compounds (refer Table 2 for sample compositions).

The alteration in the cure characteristics of these compounds must be meticulously assessed in relation to parameters such as *M*_H_ − *M*_L_ (Δ*M*), cure rate index (CRI), scorch time (*t*_s2_), and optimum cure time (*t*_90_), since the modification in one parameter may be elucidated by another. The curing behavior of the prepared rubber compounds was evaluated using a rheometer, and the resulting torque–time curves are presented in Fig. 4, while the corresponding curing parameters, including *M*_H_ − *M*_L_ (Δ*M*), CRI, *t*_s2_, and *t*_90_ are summarized in Table 5. The results indicate that the incorporation of DE with silica will not adversely impact the curing characteristics, as sustaining an optimal Δ*M* while achieving a reduced *t*_90_ is crucial for industrial procedures. The Δ*M* value indicates the crosslink density of vulcanizates. The findings indicated that silica had the highest Δ*M* value hence the highest cross-linking density. It has been found that silica does not obstruct the spaces between rubber molecules, facilitating crosslinking.^26^ Additionally, during the mixing process, significant abrasion occurs, resulting in a reduction in silica particle size. This reduction enhances heat generation while the vulcanization at 150 °C within the composite, which in turn promotes increased cross-link formation. Even though the SEM and DLS findings support the argument that silica has a larger particle size compared to DE, the larger particles depicted in Fig. 1(b) will fragment into smaller primary particle sizes during the mixing process. Despite the apparent size of silica particles, their actual dimensions range from 80 to 90 nm. In rubber composites, silica or predominantly silica-based fillers demonstrate micro-dispersion with particle sizes not exceeding 100 nm when adequate coupling agents are present in the composite. However, this does not apply to DE, which may encompass particle sizes from 2 to 300 μm. The reinforcement of the composite may also be detrimentally affected by the low density of DE. The bulk density of silica is approximately 2.23 g cm^−3^, but DE ranges from 0.51 to 0.55 g cm^−3^,^8^ indicating that a larger volume of DE is incorporated into the rubber compound. The increased volume further contributes to the agglomeration process in the rubber matrix.

**Table 5:** Variation of *M*_H_ − *M*_L_, *t*_s2_, *t*_90_, and CRI of prepared rubber compounds

	Silica	25DE	50DE	75DE	DE
*M*_H_ − *M*_L_ (dNm)	18.445	17.045	17.195	16.765	12.165
*t*_S2_ (min)	1.7	1.71	2.05	1.63	2.62
*t*_90_ (min)	10.82	7.17	8.52	8.19	13.18
CRI (min^−1^)	13.29	18.31	15.45	15.24	9.47

Table 5 also illustrates the correlation between the CRI and different rubber compounds. CRI typically indicates the rate of the vulcanization process. This may be modified by the accelerator, activator, vulcanizing system, presence of impurities, and thermal conductivity. Notably, DE filled rubber compounds have a significantly low CRI. As DE possesses nano-scale pores, these pores can capture sulfur particles, thereby inhibiting the synthesis of cross-link precursors due to a deficiency of sulfur and accelerators necessary for cross-link formation. This also supports that DE-filled rubber compounds exhibit a lower Δ*M* (*M*_H_ − *M*_L_) than silica-filled compounds, indicating a lower crosslink density of the vulcanizates. However, silica-based fillers exhibit significantly lower thermal conductivity compared to carbon black (CB).^27^

The *t*_s2_ value, representing the scorch time or the onset of vulcanization, is comparatively low for silica. This suggests that cross-linking in the silica-filled composite begins earlier than in other composites. Silica appears to have a larger particle size compared to DE, but during milling, these aggregates can break down into finer particles. Smaller particles within the rubber matrix increase the surface area available for interaction with rubber molecules. This enhances molecular mobility and accelerates heat generation due to increased abrasion, promoting earlier cross-link formation and reducing *t*_s2_.

The increased surface area of these smaller particles also facilitates a higher number of cross-links per unit volume since smaller filler particles allow the rubber molecules to locate much closer to each other, leading to an elevated cross-link density. Consequently, *t*_90_, the time required to achieve 90% of total cure, of the 100% silica composite is extended. However, this prolonged curing rate may also indicate complexities in the dispersion of silica or interaction dynamics within the matrix. In contrast, the *t*_s2_ and *t*_90_ values for the 100% DE composite indicates a notably slower initiation of cross-link formation, producing a more pronounced curing curve. DE particles appear to hinder the production of cross-link precursors, thereby delaying the curing process. This suggests that the particle characteristics of DE reduce the efficiency of cross-linking reactions within the rubber matrix. Nevertheless, as shown in Table 5, DE-filled rubber compounds can still be fully vulcanized within 13 minutes at 150 °C, which is within acceptable industrial limits, despite their slightly slower vulcanization rate compared to silica-filled compounds.

**Fig. 5:**
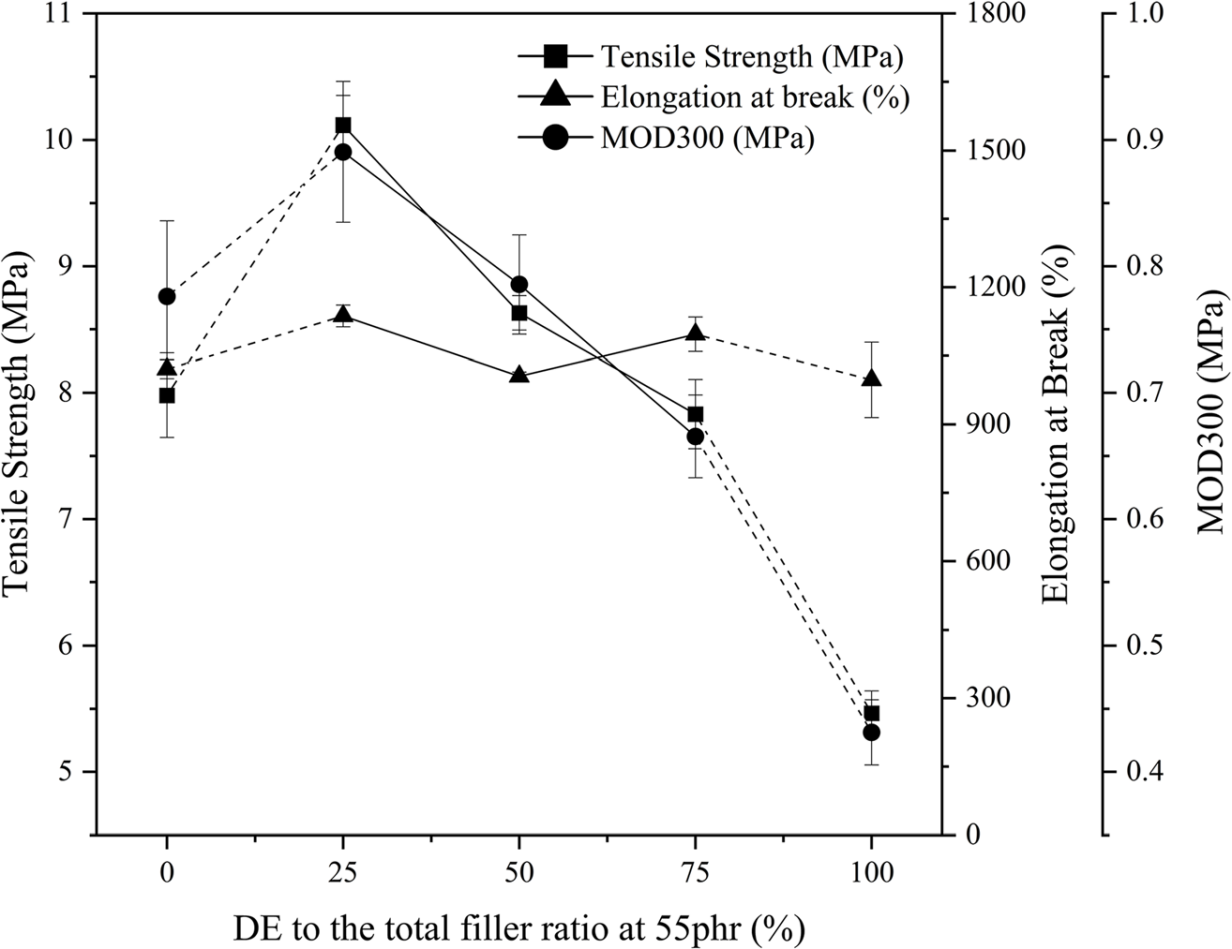
Variation of tensile strength, elongation at break, and MOD300 with DE to the total filler ratio at 55 phr*. * Note that the variation of properties from 0–25 and from 75–100 might exhibit a notable variation. As it is not validated using experimental data, it has been denoted using a dotted line.

**Fig. 6:**
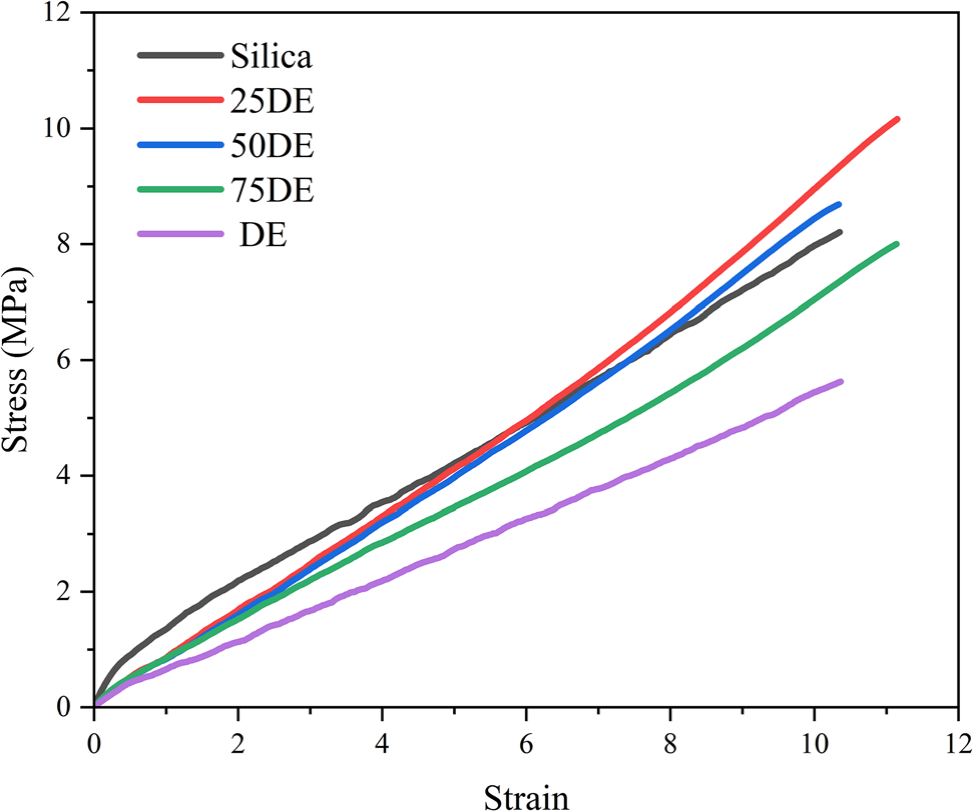
Stress *vs.* strain curves for the composites (refer Table 2 for sample compositions).

The primary tensile properties were analyzed to identify the rubber composite that exhibits the most promising characteristics. Fig. 5 illustrates the variation of tensile strength, elongation at break, and the modulus at 300% elongation (MOD300) of these composites while Fig. 6 illustrates the stress *vs.* strain curves for the composites.

The most notable observation is that the composite containing 25% DE exhibits the highest tensile properties, as shown in Fig. 5. This enhancement can be attributed to the high porosity of DE, which facilitates greater filler-rubber interactions *via* the coupling agent, owing to an increased exposure of silanol groups. These interactions strengthen the vulcanizate through improved interfacial bonding. The elevated tensile strength is the result of synergistic effects involving enhanced filler–matrix interactions, increased cross-link density, and improved compatibility. However, beyond this optimal loading, a further increase in DE content leads to a decline in tensile properties. This is partly due to DE's significantly lower density compared to silica, which limits its reinforcing capability under high-stress conditions. Additionally, excessive filler content promotes particle agglomeration, reducing the efficiency of stress transfer and thereby compromising the mechanical performance of the composite.

While the elongation at break for all composites falls within the range of 1000–1300%, the tensile modulus follows a similar trend, indicating that elongation behavior is largely governed by the material's ability to transmit stress. When the filler exhibits optimal interfacial contact, sufficient strength, and uniform dispersion within the matrix, stress transfer is efficient, allowing the rubber chains to elongate without premature failure. However, the presence of filler agglomerates disrupts this interaction, reducing effective filler–matrix contact and leading to the rupture of filler domains under strain. No consistent trend is observed in elongation with increasing DE content. Although higher DE loading may promote agglomeration, the size and distribution of these agglomerates are difficult to quantify, making their influence on mechanical properties less predictable.

## 4 Conclusions

DE is a silica-based material known for its versatility and potential to replace conventional silica with a wide range of applications. In this study, it was initially found out that amorphous DE contains 92.5% silicon by weight of the total detected elements. This DE was partially substituted for silica in rubber composites at a loading of 55 phr. Notably, the rubber composite with a 25% DE substitution demonstrated rheological parameters comparable to those of composites containing 100% commercial silica, while exhibiting superior tensile properties.

These findings highlight the potential of DE as a cost-effective and naturally occurring alternative to synthetic silica in rubber formulations. The improved mechanical properties suggest that DE-filled rubber composites could be advantageous in various applications, including the production of automotive components, industrial seals, gaskets, footwear soles, and vibration-dampening materials. Moreover, as a material derived from natural bio-origin deposits, the use of DE may contribute to developing more environmentally conscious rubber products by reducing dependency on synthetic fillers. Future research could explore optimizing the filler content and evaluating long-term durability for more extensive industrial applications.

## Author contributions

N. B. J. De Costa conducted the majority of the experimental work and was primarily responsible for manuscript preparation. S. M. Egodage contributed her expertise in rubber processing, cure characteristic analysis, and mechanical property evaluation. M. Maddumaarachchi served as the principal investigator of the project, providing overall supervision and guidance. All authors reviewed and approved the final manuscript.

## Conflicts of interest

On behalf of all authors, the corresponding author states that there is no conflict of interest.

## Data Availability

The data used to support the findings of this study are included within the article.
